# Establishing the feasibility of exercise breaks during university lectures

**DOI:** 10.3389/fspor.2024.1358564

**Published:** 2024-04-04

**Authors:** Scott M. Hayes

**Affiliations:** ^1^ Department of Psychology, The Ohio State University, Columbus, OH, United States; ^2^ Chronic Brain Injury Initiative, The Ohio State University, Columbus, OH, United States

**Keywords:** acute exercise, exercise, attention, mind wandering, memory, teaching, cognition, academic performance

## Abstract

It is important that principles of laboratory-based studies with implications for academic performance be implemented in naturalistic learning environments to gauge their feasibility. Here, an adaptation of a laboratory-based study of exercise breaks during a single video lecture was implemented during large, in-person lectures at Ohio State University for the duration of a semester. The rationale for this approach was based on findings that research participants who took exercise breaks during a video lecture were more likely to be on task towards the end of the lecture and performed significantly better on a multiple choice exam. The current project had three goals: (1) Establish the feasibility of integrating student-led exercise breaks during in-person lectures in a large university setting (2) Provide practical guidelines for implementing exercise breaks during in-person lectures (3) Provide preliminary evidence of positive effects of exercise breaks in a higher-education setting. One to two student-led exercise breaks (5 min each) were implemented during each 80 min, in-person lecture for the duration of a semester in four upper level Psychology courses with student enrollment ranging from 20 to 93 students (total enrollment = 223 students). Students reported that the exercise breaks were a strength of the courses and a positive experience, including self-reported improvement in attention to lecture content. Self-reported quantitative data indicated that exercise breaks improved attention, increased course enjoyment, and enhanced peer engagement. Compared to other classes, the students preferred exercise breaks during lectures. The current approach establishes the feasibility of integrating exercise breaks in a large, in-person university lecture environment for the duration of a semester with preliminary data indicating a positive impact on attention, engagement, and enjoyment. Practical guidelines for implementing exercise breaks during in-person lectures are provided.

## 1 Introduction

There is a great deal of interest in cognitive enhancement. One of the more promising methods for optimizing cognitive performance is exercise, which has demonstrated positive effects on executive functions and memory (1, 2) as well as brain structure and function (3–5). Examination of the acute effects of exercise on cognition has occurred in two broad domains: controlled laboratory settings or naturalistic classroom settings. Studies in laboratory settings have (1) tended to use cognitive tests as the outcome measure (2) tested undergraduate students (age = 18–23 years) and (3) used an aerobic exercise stimulus (cycling, treadmill running). A meta-analysis of predominately laboratory studies showed a small but positive effect of acute exercise on executive function (attention, planning, and inhibition (6) and a recent study indicated that a 10 min bout of exercise may enhance memory (7).

Classroom-based exercise studies have typically assessed children and adolescents, and incorporated a range of outcome measures, including cognition, classroom behaviors, or academic performance (8). These studies, the majority of which used aerobic exercises, have reported positive effects on cognition (9). However, one meta-analysis (8) did not report beneficial effects of exercise on cognition or academic performance, but exercise was associated with better classroom behavior. A limitation of the meta-analysis was the small number of studies (*n* = 8) within a given intervention type or outcome measure. Nevertheless, time on task was improved in most studies and the relationship was dose-dependent based on exercise volume.

Given the absence of studies of exercise interventions in collegiate classrooms, there is limited guidance on best practices to optimize attention and classroom performance in young adults. However, a recent laboratory study by (10), who implemented prescribed 5 min aerobic exercise breaks in undergraduates during a single videotaped lecture, provides a conceptual foundation. The authors implemented three, 5 min aerobic exercise breaks throughout a single 50 min videotaped lecture. Compared to students who played a video game or had no break (traditional lecture format), the exercise group was more likely to be on-task towards the end of the lecture. The exercise group also exhibited better memory performance on an immediate and a delayed multiple-choice exam and provided superior ratings on narrator clarity and their own understanding of the materials.

During in-person university lectures, students are highly sedentary, typically sitting for the duration of the class (55–80 min). Furthermore, as time on task increases, such as listening to a lecture, mind wandering increases and memory for lecture content decreases (11, 12). These findings, coupled with those reported by Fenesi et al. (10), highlight the need to establish the feasibility of implementing an exercise protocol during live, in-person lectures in a university classroom. To address this issue, exercise breaks were implemented during four in-person courses at The Ohio State University (Columbus campus: undergraduate enrollment ∼46,000; graduate and professional student enrollment ∼6,300). Course lectures were 80 min each, twice per week, for the entire 15-week semester. In addition to potential cognitive benefits of improved attention and memory, inserting bouts of exercise during the lecture provided an opportunity to improve mood as well as model healthy behavior to students, i.e., avoid being sedentary, and challenge narrow definitions of physical activity, e.g., “I need to dedicate one hour to exercise at the gym 3–5 times per week.” This activity also provided an opportunity for students to interact in smaller groups. There were three main goals: (1) Demonstrate the feasibility of implementing exercise breaks in a large, in-person lecture setting (2) Describe practical guidelines for implementing exercise breaks during in-person lectures (3) Provide preliminary evidence of positive effects of exercise breaks in a higher-education setting.

## 2 Pedagogical framework and methods

Students earned class points for designing the exercise routine, serving as group leaders, and for participating in exercise breaks led by other student groups. Below is a brief description of the approach (Table 1).

(1) Students from the larger classes (70 or more students) were randomly assigned to groups of six or seven students. For the smaller classes (less than 40 students), group size was two to three students. The number and size of the groups were selected so that each group led an exercise routine for at least one class during the semester. To determine group size, one can divide the number of students in the class (e.g., 100) by the number of classes in the semester (e.g., 20; do not count classes on which there will be an exam). This example would result in a group size of 5 students, with each student group leading an exercise break once during the semester. For smaller classes, one can select a group size of two to four students, and they can lead exercise breaks multiple times during the semester.
(2) The Fenesi et al. (10) article was assigned as a required reading for the first class, which provided the scientific rationale (improved attention, better exam performance, and improved narrator quality) for in-class exercise breaks. During the first class, the study was discussed in detail. Primary points of emphasis included discussion of the experimental group assignment (exercise group vs. video game playing group vs. lecture as usual), tasks completed during the break (e.g., specifics of the exercise intervention, video game played), and main study findings (students in exercise group were more likely to be on task, gave higher narrator quality ratings, and had higher multiple choice exam scores compared to the video game playing group and the lecture as usual group). Reading and discussion of the article occurred to provide context and understanding to the students; in other words, the answers to why we would be taking exercise breaks during the class. It was the author's opinion that this was important to get “buy-in” from the students, all of whom had taken dozens of courses at the university level and had never taken an exercise break during a lecture.
(3) During the first class, the Physical Activity Readiness Questionnaire (PAR-Q & You) was presented and discussed. The PAR-Q is a brief preparticipation health screening form recommended by the American College of Sports Medicine and was to assess whether someone may safely initiate becoming more physically active. The PAR-Q was administered to confirm safety of student participation in the exercise breaks. The PAR-Q was recently updated (PAR-Q+) and it is recommended that the latest available version be used for assessment (13).
(4) After the first class, the PAR-Q was posted on-line for students to complete before the third class (when the exercise breaks would start). A small number of students (roughly 5%) responded “yes” to at least one of the questions. In these cases, the instructor spoke or emailed with them individually about the exercise breaks. The students were instructed to avoid any exercises that could exacerbate their condition. They were also given permission not to participate or stay in their seats, to stand, stretch, modify the prescribed exercise, choose a different exercise, or leave the room for a brief break. All students elected to participate in the exercises and modified or chose alternative exercises as needed.
(5) For the second class, students met with their groups. The students were assigned multiple tasks:
  a. Generate a group name [e.g., Freudian Dipz, Forever Jung, The Control Group, Scott's Tots (“The Office” reference)]. This was implemented as an ice-breaker and a low-stakes, creative group activity for the students.
  b. Learn how to calculate exercise intensity based on their heart rate (HR). This entailed teaching students how to calculate their age-predicted heart rate max (here we used the simple equation, heart rate max = 220-age), take their own pulse (carotid or wrist or use their smartwatch or activity tracker) and then calculate % HR max by dividing HR current/HR max. Data from Fenesi et al. (10) demonstrated that participants reached about 70% of their HR max during their prescribed routine. The class was instructed to target 50%–70% HR max, which would fall within the light to moderate intensity range (14). Rationale for this range was based on the following: (1) no definitive study showing whether various exercise intensity levels confer differential cognitive benefits in an academic setting (2) practical aspect of avoiding excessive perspiration by exercising at a vigorous intensity for the duration of the break.
  c. Create an exercise routine: 5 exercise bouts, 50 sec each, with a 10 sec break between each bout. The number of bouts and timing was modelled after (10). For the exercise routine, students had the freedom to implement a mix of exercises (aerobic, strength, flexibility, balance, yoga poses, or deep breathing) with the caveat that at least three needed to be aerobic. For 50 sec bouts that were targeted as flexibility or stretching, students were instructed to generate two to three different stretches per bout. If at least three of the five exercises were aerobic, this would be sufficient to elevate heart rate for the majority of the 5 min exercise period (which the students anecdotally confirmed in class when heart rate was self-assessed during the first session). A variety of exercises was preferred to maintain student engagement throughout the semester (Table 2).
  d. Confirm that their exercises were feasible to implement in the classroom.
  e. Post the exercise routine to online discussion group. This provides the instructor the opportunity to review the exercise routine and provide feedback. For instance, if a group had chosen five lower body aerobic exercises, suggestions were made to vary the routine.
(6) On the third class and thereafter, the exercise breaks were implemented for each lecture. At the beginning of class, the instructor announced the student group leading exercises. At around the 25 min mark of class, the group was invited to the front of the auditorium. Each group member introduced themselves. The group was responsible for demonstrating the exercises to the class, timing the duration of each exercise, and timing the 10 sec breaks between exercises. Student groups also had the option to play music, and most groups elected to play music from a smartphone which was broadcast to the class via the lecturer's microphone. After the exercise break, the group leaders and the students returned to their seats. The lecture continued with another exercise break around the 55 min mark of the class. For classes in which one exercise break was implemented, the break occurred at the 40 min mark of the 80 min class.
(7) The instructor participated and completed the exercise bouts with the students. Occasionally, the instructor intentionally deviated from the prescribed exercises, further reinforcing that exercises can be modified as needed.

**Table 1:** Summary of protocol for implementing exercise bouts during in-person lecture.

• Assign and discuss Fenesi et al. (10) article with the class
• Students complete Physical Activity Readiness Questionnaire
• Student groups meet and generate 5 min exercise routine
• Instructor reviews proposed exercise routines for safety and practicality of implementation
• Each class thereafter, student groups lead all exercise breaks with support from instructor

**Table 2:** Examples of student generated exercise routines with 10 s rest between each bout.

Routine	Bout 1	Bout 2	Bout 3	Bout 4	Bout 5
1	Jumping Jacks	Deep breathing and hand stretches	Butt kickers	Side-stance leg stretch	High knees
2	Alternating hamstring stretch (left, right, center)	Jump squats	Reverse lunges	High knees	Deep breathing
3	Line Jumps (25 s forward/backwards and 25 s side to side)	Stretching: Interlock hands behind back and lift; standing shoulder rotation stretch; standing cross-body stretch	Overhead press (with backpack)	Heel sweeps; standing quadriceps stretch; lunge stretch	6 squats and then 6 lunges–repeat

### 2.1 Qualitative data collection

All four courses provided an opportunity for students to provide on-line anonymous comments about the course at the end of the semester. These student evaluation of instruction forms are automatically offered for all students taking courses at Ohio State University. Exercise breaks are not specifically probed in these standard evaluations, but there is an open field for students to comment (positively or negatively) about the class. The evaluations were examined for both positive and negative comments about the exercise breaks. Two classes (SP 2020 Health Psychology and SP 2020 Cognitive Aging) also had a PhD-level teaching consultant (outside of the instructor's home department and separate from the instructor peer-review process) attend a class to collect student impressions of the course (strengths and weaknesses) and provide anonymous feedback to the instructor. Pre-tenure faculty are also observed annually by tenured peers within the department. The peer review letters were available for two courses and were also used as a source for qualitative assessment of exercise breaks.

### 2.2 Quantitative data collection

At the end of the spring 2023 Cognitive Aging course, 27 of the 37 enrolled students completed a voluntary and anonymous on-line 7-item survey with a 5-point Likert scale plus an additional open-ended item that probed for general comments about the exercise breaks (“Please provide any other feedback/comments related to your impression of the exercise breaks.). One-sample t-tests were implemented as an item analysis to assess whether subjective ratings were significantly different from 3, which corresponded to a rating of “neutral” on the Likert scale.

## 3 Learning environment

This protocol was designed to be implemented in a higher education setting with adults. The exercise breaks described above were implemented in large lecture halls with movie theater style seating, as well as active learning classrooms with students seated in smaller groups of four to six students around a table. In the current study, the exercise breaks were implemented for the semester in four upper-level Psychology courses: Spring 2019 Health Psychology (enrollment = 93 undergraduates); Spring 2020 Health Psychology (enrollment = 73 undergraduates); Spring 2020 Cognitive Aging (enrollment: 13 undergraduates; 7 graduate students); Spring 2023 Cognitive Aging (enrollment: 32 undergraduates, 5 graduate students). The gap in implementation from 2020 to 2023 was due to asynchronous, on-line course offerings due to COVID-19. Each course met twice per week for 80 min for the duration of the semester, although one health psychology course and one cognitive aging course met for 10-weeks (in-person classes were cancelled in the latter part of the 2020 semester due to COVID-19). At least one student-led exercise break was implemented for every lecture.

## 4 Results

### 4.1 Qualitative data

The overall impression from the qualitative data was that the students self-reported benefits from the exercise breaks, including improved motivation, attention, and engagement with peers and the instructor. For instance, although exercise breaks were not specifically probed, students spontaneously provided anonymous comments with their end-of-semester student evaluation of instruction (SEIs). Comments included the following:

“I enjoyed the exercise breaks in class and really felt like they motivated me to focus more.”

“I love that the instructor puts research into practice in his classroom with the exercise breaks! I found these really fun… They also helped a lot with my focus during a long lecture.”

“Something I really enjoyed about this class were the exercise breaks given in class. They really did help break up some of the material and made staying engaged in class easier.”

There were a handful of negative comments about the exercise breaks on the end-of-semester student evaluations of instruction. One student described dreading the exercise breaks, and another suggested that one exercise break, rather than two, would be sufficient.

The positive comments were consistent with those reported in the end of semester survey for the spring 2023 cognitive aging course. Of the 27 students who completed the survey, 16 elected to provide general feedback about the exercise break. The unedited student feedback from all students is reported in Table 3. Most student responses described a positive impact of exercise breaks on their lecture experience. Many of the comments reported positive effects on attention and class enjoyment.

**Table 3:** Unedited student comments from survey offered in spring 2023 cognitive aging course.

This was a great addition to the class! I enjoyed the combination of aerobic and anaerobic exercises.
They were fun! For me, it strengthened my relationship with my peers and the professor allowing me to be more comfortable in class. It eliminated any awkwardness that comes from being in a class of strangers.
I think it was a great way to refocus attention
Referring to Question 8, it doesn't have to be student-led, but even a stand up and stretch or water/walking break would be amazing! In 5089, the exercise breaks were enjoyable, but for many of the other larger classes the latter would probably be easier and still very helpful as well. Thank you!
The exercise breaks helped wake me up and were a nice way to break up the lecture period. Sitting there and getting talked at for 80 min is pretty draining, and I thought the exercise breaks did an awesome job of keeping me engaged and alert during class. I would really like to see this incorporated into more classes, especially since physical movement has been shown to have big benefits for students (treadmill/cycle desks, standing desks, etc.).
I like the idea of exercise breaks however I felt the groups who incorporated meditation into their breaks had an overall better effect. After the meditation breaks, I felt more centered and more present in class. I think by incorporating both into the breaks can help students get moving but also feel more mindful and connected to the class.
It really helped to be able to section the class into two sections. I felt more aware during the second presentation. Not sure if I would be able to focus as well during the second presentation without the exercise break.
It might be more fun if all of the exercise breaks had themes rather than just normal exercises and rest periods with music.
I really enjoyed the exercise breaks! Several groups had controlled breathing exercises in between sets of aerobic exercises, which maybe should be avoided just to avoid a rare possibility of fainting or panic attacks in participants.
I feel like the exercise breaks helped me get to know my classmates better, and it was a nice break from the lectures. It was also a good transition between the presentations.
I thought the exercise breaks were an enjoyable way to refocus in the middle of class. Sometimes, I wished the exercise breaks were a bit more vigorous with less emphasis on stretching and more on aerobic exercises. I think the music also makes the breaks more enjoyable. Overall, I think it would be good to continue implementing exercise breaks during class.
I liked when there were options to make each exercise more or less strenuous.
I thought the exercise breaks were enjoyable overall, and I liked the idea behind them, but subjectively I don't feel as though they improved my focus or attention in lecture.
The exercise break was a good way to split up the class and maintain my attention. I find it hard to sit through an hour 20-minute lecture and stay completely focused the whole time.
They were good.
The exercise breaks helped break up the class. It was nice being able to get up instead of sitting for 80 min.

Additional qualitative evidence of the positive impact of exercise breaks on student's subjective lecture experience were confirmed with another reporting modality for collecting anonymous student feedback: a PhD-level teaching consultant attended two classes and solicited anonymous feedback from students (without the instructor present). During the session, the consultant posed the following question: “What are the strengths of the course and instructor that assist you in learning?” For the spring 2020 health psychology course, eight of the 12 student groups spontaneously listed the exercise breaks as one of the strengths (note that they were not specifically asked about the breaks but listed it as a strength nonetheless). For the Cognitive Aging course, although only two of the five groups spontaneously listed exercise breaks as a strength, the independent teaching consultant documented it as a general strength because “most students” liked the exercise breaks. Student group comments included “We like the exercise breaks. It helps us stay focused” and “Exercise breaks are refreshing.”

Finally, at Ohio State University, professors are peer-reviewed while teaching. In the formal course peer evaluation, a tenured colleague noted “Something quite remarkable occurred approximately halfway through the lecture…the instructor announced “Exercise break!” and—to my amazement—the students stood up and started doing jumping jacks, squats, and other physical exercises…For the record, so was I and I can attest from personal experience that the exercises had an extremely beneficial effect on my ability to listen attentively when the lecture resumed.” Two colleagues within the Psychology Department have reported that they have subsequently initiated exercise breaks in their courses.

### 4.2 Quantitative data

**Figure 1:**
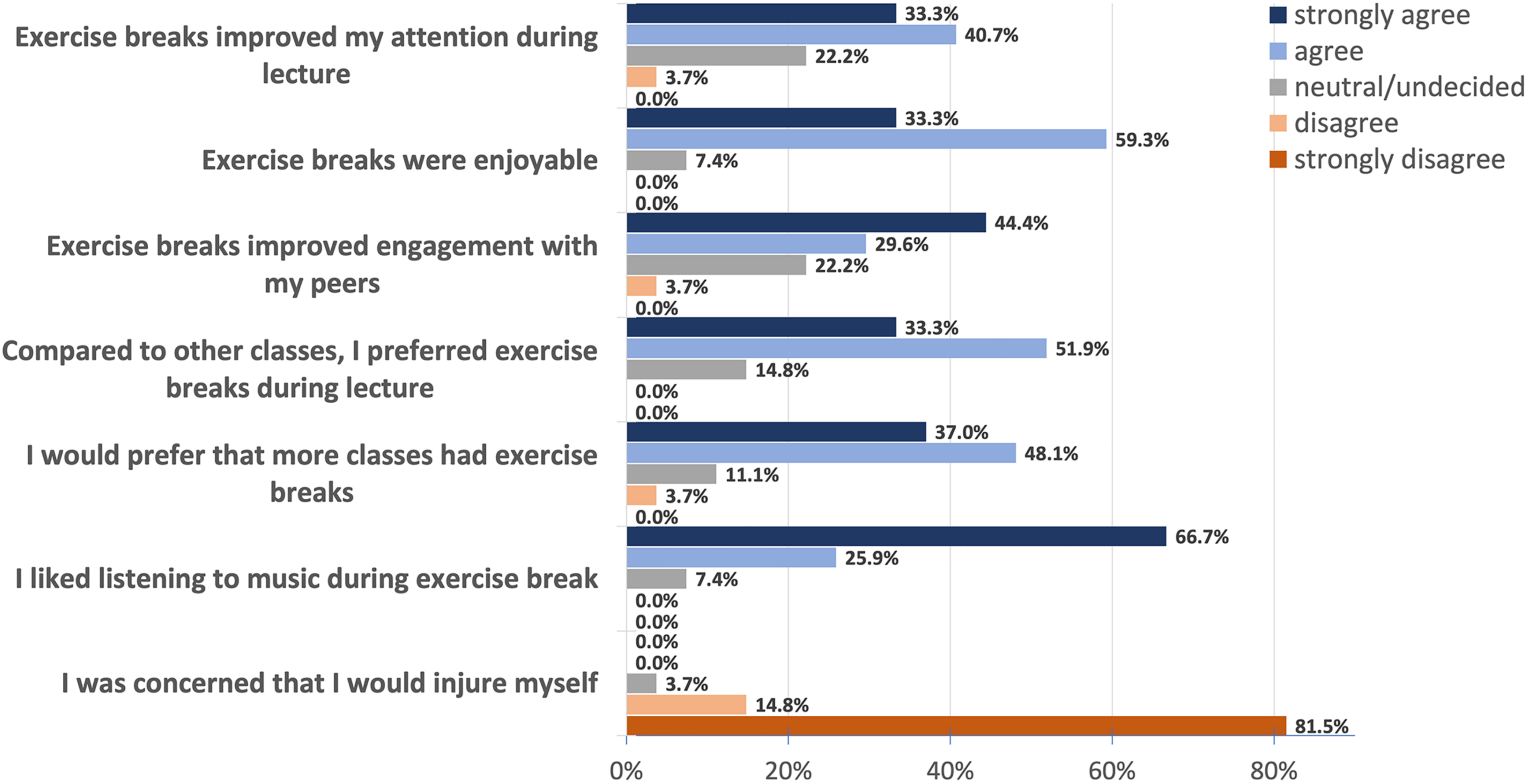
Percentage of students selecting response options for survey items in spring 2023 cognitive aging course.

All students reported that they had never taken a class that implemented an exercise break during a lecture. The results of the self-reported quantitative data were consistent with the qualitative data (Figure 1). One-sample *t*-tests (test sample value = 3, corresponding to a “neutral rating”) revealed that students rated the exercise breaks as improving attention, enjoyable, and improving peer engagement, all t's (26) > 6.30, all p's < .001 (Table 4). The students reported that compared to other classes, they preferred exercise breaks during the lecture and that they would prefer that more classes offered exercise breaks t's (26) > 7.82, all p's < .001. Students reported that they preferred listening to music during the exercise break. Students were not concerned about injury (Figure 1; Table 4). All results remained significant after Bonferroni multiple comparisons correction (*p* = 0.05/7 = .007).

**Table 4:** Mean, standard deviation (sd), and results of one-sample *t*-tests for each item [*t*-test value = 3 (neutral), two-tailed significant test].

	Mean (sd)	t-statistics
Exercise breaks improved my attention during lecture	4.04 (0.85)	t (26) = 6.31, *p* < .001
Exercise breaks were enjoyable	4.26 (0.59)	t (26) = 11.01, *p* < .001
Exercise breaks improved engagement with my peers	4.15 (0.91)	t (26) = 6.58, *p* < .001
Compared to other classes, I preferred exercise breaks during lecture	4.19 (0.68)	t (26) = 9.04, *p* < .001
I would prefer that more classes had exercise breaks	4.19 (0.79)	t (26) = 7.83, *p* < .001
I liked listening to music during exercise break	4.59 (0.64)	t (26) = 13.01, *p* < .001
I was concerned that I would injure myself	1.22 (0.51)	t (26) = −18.24, *p* < .001

Response options: 1 = strongly disagree, 2 = disagree, 3 = neutral, 4 = agree, 5 = strongly agree.

## 5 Discussion

The current report establishes the feasibility of implementing student-lead exercise breaks during in-person lectures with undergraduate and graduate students in a university setting. The exercise breaks were implemented across four courses in active-learning classrooms with a class size as small as 20 students as well as larger lecture halls with movie theater style seating with 93 students. Qualitative and quantitative self-reported data were consistent: students reported positive impacts of exercise breaks on attention and motivation, engagement with their peers (which is often difficult to establish in large classes), and course enjoyment. Finally, the current report provides a protocol for implementing exercise breaks in a university setting.

Acute bouts of exercise exert multiple influences on various domains, including emotion, cognition, and physiology that are likely contributing to the students’ reported positive impacts of exercise breaks during university lectures. For instance, acute bouts of exercise have a positive impact on depression and hostility (15), anxiety (16), and affect and energy (17). Further, in addition to the Fenesi et al. (10) study, other laboratory studies have demonstrated a positive impact of acute bouts of exercise on cognition, including executive function and memory (6, 18–22). These positive benefits of exercise on mood and cognition are likely underpinned by the cascade of physiological alterations observed subsequent to an acute bout of exercise, including elevated heart rate, alterations in levels of cortisol, endorphins, neurotransmitters, and neurotrophins, and alterations in neuronal firing and cerebral blood flow (for review, see (18).

Anecdotally, throughout the semester, multiple students openly commented about how much they liked the exercise breaks, and it appeared that all students in any given class participated. One student noted it had motivated them to initiate exercise routines outside of the classroom. At a broader level, implementing exercise breaks during lectures provides an opportunity for instructors to model positive health behaviors, as multiple lab-based studies have reported positive associations between physical fitness, physical activity, cognition, and the brain (5, 23, 24).

It is important to recognize that the exercise bouts should be implemented thoughtfully and in an organized manner at the start of the semester. One should consult with knowledgeable professionals (colleagues in physical education, exercise science, or physical rehabilitation) if unsure about the practicality of an exercise or concerned about potential physical limitations of students. It is important to emphasize that both the instructor and students use common sense when thinking about the exercise routine and an individual’s exercise abilities and err on the side of caution. Most undergraduate and graduate students are younger adults, and the risk for an adverse event is extremely low. Fewer than 5% of students reported an issue on the PAR-Q, and these were predominately acute injuries (e.g., sprained ankle). Moreover, the exercises should fall within the intensity range of normal activities of daily living, further mitigating risk of adverse events. Nevertheless, some students have limitations, and the burden of inclusion in this activity falls upon the instructor and appropriate collaboration with the students.

The anecdotal nature and potential biases of these data sources are acknowledged; nevertheless, these data provide insight into the tolerance and feasibility of implementation of exercise breaks in a higher education setting and indicated multiple positive effects of exercise among students. Future studies could implement more detailed assessment or assess tolerance in other undergraduate samples or courses (beyond psychology and neuroscience). More rigorous academic assessment can be challenging to implement in a typical university class setting for the duration of the semester. One could implement an ABA design (no exercise, exercise, no exercise) during the semester, although one would need to control for course content if attempting to assess academic performance, comprehension, or memory for the materials. Similarly, one could attempt to compare exam performance for different offerings of the course with and without exercise breaks, and attempt to address the challenges of cohort effects, time of day effects, and instructor effects.

For the instructor, there were some self-reported beneficial effects from the exercise breaks. The bouts of exercise provided a brief respite from speaking, naturally introduced variability in the lecture, and reinvigorated the instructor for the next bout of lecture. It also provided a unique opportunity to get to know the students and served as a collaborative group activity for students to get to know their peers, which is often difficult to achieve in a course with many students.

Finally, these data warrant consideration for implementation of exercise breaks in more courses at the University level. Given that students typically sit for the duration of the lecture, exercise breaks reduce sedentary behavior, which is associated with negative health outcomes and may negatively impact cognition and brain health (25, 26). Moreover, these data indicate that implementation of exercise breaks throughout the semester is feasible. The exercise breaks were acceptable and well-tolerated by the students. Students perceived positive impacts on attention, peer engagement, enjoyment of the exercise breaks, and preferred the exercise breaks during the lecture. As a practical, albeit ambitious example, it is worth noting that a student taking a 12-credit semester course load (4 courses × 2 classes per week × 5 min of exercise per class = 40 min of moderate exercise per week) would reach 26% of the weekly recommended goal of 150 min of moderate physical activity per week (14) by participating in the exercise breaks during their classes. Thus, larger scale adoption of these healthy behaviors, such as exercise breaks during lectures, have the potential to contribute to weekly physical activity recommendations and associated positive mental and physical health outcomes in students attending university.

## Data Availability

The original contributions presented in the study are included in the article/Supplementary Material, further inquiries can be directed to the corresponding author.

## References

[1] Colcombe S Kramer AF. Fitness effects on the cognitive function of older adults: a meta-analytic study. Psychol Sci. (2003) 14(2):125–30. 10.1111/1467-9280.t01-1-01430 12661673

[2] Kramer AF Colcombe S. Fitness effects on the cognitive function of older adults: a meta-analytic study-revisited. Perspect Psychol Sci. (2018) 13(2):213–7. 10.1177/1745691617707316 29592650

[3] Esteban-Cornejo I Rodriguez-Ayllon M Verdejo-Roman J Cadenas-Sanchez C Mora-Gonzalez J Chaddock-Heyman L Physical fitness, white matter volume and academic performance in children: findings from the ActiveBrains and FITKids2 projects. Front Psychol. (2019) 10:208. 10.3389/fpsyg.2019.00208 30809168 PMC6379335

[4] Hayes SM Hayes JP Cadden M Verfaellie M. A review of cardiorespiratory fitness-related neuroplasticity in the aging brain. Front Aging Neurosci. (2013) 5:31. 10.3389/fnagi.2013.00031 23874299 PMC3709413

[5] Prakash RS Voss MW Erickson KI Kramer AF. Physical activity and cognitive vitality. Annu Rev Psychol. (2015) 66:769–97. 10.1146/annurev-psych-010814-015249 25251492

[6] Chang YK Labban JD Gapin JI Etnier JL. The effects of acute exercise on cognitive performance: a meta-analysis. Brain Res. (2012) 1453:87–101. 10.1016/j.brainres.2012.02.068 22480735

[7] Suwabe K Hyodo K Byun K Ochi G Yassa MA Soya H. Acute moderate exercise improves mnemonic discrimination in young adults. Hippocampus. (2017) 27(3):229–34. 10.1002/hipo.22695 27997992 PMC5927776

[8] Daly-Smith AJ Zwolinsky S McKenna J Tomporowski PD Defeyter MA Manley A. Systematic review of acute physically active learning and classroom movement breaks on children’s physical activity, cognition, academic performance and classroom behaviour: understanding critical design features. BMJ Open Sport Exerc Med. (2018) 4(1):e000341. 10.1136/bmjsem-2018-000341 29629186 PMC5884342

[9] Kubesch S Walk L Spitzer M Kammer T Lainburg A Heim R A 30-minute physical education program improves students’ executive attention. Mind Brain Educ. (2009) 3(4):235–42. 10.1111/j.1751-228X.2009.01076.x

[10] Fenesi B Lucibello K Kim JA Heisz JJ. Sweat so you don’t forget: exercise breaks during a university lecture increase on-task attention and learning. J Appl Res Mem Cogn. (2018) 7:261–9. 10.1016/j.jarmac.2018.01.012

[11] Farley J Risko EF Kingstone A. Everyday attention and lecture retention: the effects of time, fidgeting, and mind wandering. Front Psychol. (2013) 4:619. 10.3389/fpsyg.2013.00619 24065933 PMC3776418

[12] Risko EF Anderson N Sarwal A Engelhardt M Kingstone A. Everyday attention: variation in mind wandering and memory in a lecture. Appl Cogn Psychol. (2012) 26(2):234–42. 10.1002/acp.1814

[13] Warburton D Jamnik V Bredin S Shephard R Gledhill N. The 2021 physical activity readiness questionnaire for everyone (PAR-Q+) and electronic physical activity readiness medical examination (ePARmed-X+): 2021 PAR-Q+. Health Fit J Can. (2021) 14(1):83–7. 10.14288/hfjc.v14i1.351

[14] American College of Sports Medicine. ACSM’s Guidelines for Exercise Testing and Prescription. 11th Ed Philadelphia: Lippincott Williams & Wilkins (2021).

[15] Crush EA Frith E Loprinzi PD. Experimental effects of acute exercise duration and exercise recovery on mood state. J Affect Disord. (2018) 229:282–7. 10.1016/j.jad.2017.12.092 29329061

[16] Lucibello KM Parker J Heisz JJ. Examining a training effect on the state anxiety response to an acute bout of exercise in low and high anxious individuals. J Affect Disord. (2019) 247:29–35. 10.1016/j.jad.2018.12.063 30640027

[17] Liao Y Shonkoff ET Dunton GF. The acute relationships between affect, physical feeling states, and physical activity in daily life: a review of current evidence. Front Psychol. (2015) 6:1975. 10.3389/fpsyg.2015.01975 26779049 PMC4688389

[18] Basso JC Suzuki WA. The effects of acute exercise on mood, cognition, neurophysiology, and neurochemical pathways: a review. Brain Plast. (2017) 2(2):127–52. 10.3233/BPL-160040 29765853 PMC5928534

[19] Chang YK Tsai CL Hung TM So EC Chen FT Etnier JL. Effects of acute exercise on executive function: a study with a tower of London task. J Sport Exerc Psychol. (2011) 33(6):847–65. 10.1123/jsep.33.6.847 22262708

[20] Loprinzi PD Roig M Tomporowski PD Javadi AH Kelemen WL. Effects of acute exercise on memory: considerations of exercise intensity, post-exercise recovery period and aerobic endurance. Mem Cognit. (2023) 51(4):1011–26. 10.3758/s13421-022-01373-4 36401115 PMC9676734

[21] Waters A Zou L Jung M Yu Q Lin J Liu S Acute exercise and sustained attention on memory function. Am J Health Behav. (2020) 44(3):326–32. 10.5993/AJHB.44.3.5 32295680

[22] Weng TB Pierce GL Darling WG Voss MW. Differential effects of acute exercise on distinct aspects of executive function. Med Sci Sports Exerc. (2015) 47(7):1460–9. 10.1249/MSS.0000000000000542 25304335

[23] Hayes SM Alosco ML Hayes JP Cadden M Peterson KM Allsup K Physical activity is positively associated with episodic memory in aging. J Int Neuropsychol Soc. (2015) 21(10):780–90. 10.1017/S1355617715000910 26581790 PMC4711930

[24] Stauder M Hiersche KJ Hayes SM. Examining cross-sectional and longitudinal relationships between multidomain physical fitness metrics, education, and cognition in black older adults. Neuropsychol Dev Cogn B Aging Neuropsychol Cogn. (2023):1–15. 10.1080/13825585.2023.2225848 37345613 PMC10739568

[25] Vaynman S Gomez-Pinilla F. Revenge of the “sit”: how lifestyle impacts neuronal and cognitive health through molecular systems that interface energy metabolism with neuronal plasticity. J Neurosci Res. (2006) 84(4):699–715. 10.1002/jnr.20979 16862541

[26] Voss MW Carr LJ Clark R Weng T. Revenge of the “sit” II: does lifestyle impact neuronal and cognitive health through distinct mechanisms associated with sedentary behavior and physical activity? Ment Health Phys Act. (2014) 7(1):9–24. 10.1016/j.mhpa.2014.01.001

